# Intestinal injury and repair: insights from hyaline fibromatosis syndrome

**DOI:** 10.1038/s44321-025-00294-4

**Published:** 2025-08-22

**Authors:** Wayne I Lencer

**Affiliations:** Boston Children’s Hospital, Harvard Medical School, Boston, MA USA

**Keywords:** Digestive System, Genetics, Gene Therapy & Genetic Disease

## Abstract

In this N&V, W. Lencer discusses the study by L. Bracq and colleagues, in this issue of *EMBO Mol Med*, that used a mouse model of hyaline fibromatosis syndrome to investigate the molecular function of CMG2 in the gut.

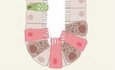

## Teaser

Polarized columnar epithelial cells form a delicate yet highly effective, single-cell-thick barrier that lines the intestine and other mucosal surfaces. Their function is to separate and actively affect vastly different physiological compartments—forming a challenging cellular interface between the host and the outside world. In the intestine, many potential dangers to cell function and survival transit the luminal gut environment—amplified by a hugely dense microbial community colonizing the mucosal surface.

The intestinal epithelium has evolved numerous mechanisms to accommodate the interface with the gut lumen. One such mechanism is the rapid and constitutive renewal of the cells that form the epithelial barrier (Gehart and Clevers, 2019; Viragova et al, 2024). Epithelial cells lining the human intestine are replaced every 5 days. These cells all originate from a small population of undifferentiated epithelial stem cells located at the base of intestinal crypts and marked by the R-spondin G-protein receptor LGR5, a key component of the Wnt-signal transduction pathway. Wnt signaling drives the cell cycle, which is characteristic of the stem cell niche (Gehart and Clevers, 2019). The LGR5+ cells give rise to progenitor cells that either replenish the stem cell population or migrate up the crypt and villus axis towards the intestinal lumen, differentiating into one of the six essential epithelial cell types needed for gut function along the way. All cells that migrate out of the crypt become increasingly exposed to the abrasive gut lumen, and within days undergo a form of non-inflammatory programmed cell death (apoptosis)—shedding into the gut lumen without disruption of the epithelial barrier.

The epithelial and non-epithelial cells that make up the stem cell niche produce factors—such as Wnt, R-spondin, and EGF—that maintain the proliferative cycle. Wnt proteins act as morphogens, affecting all aspects of embryonic development and tissue self-renewal (Gehart and Clevers, 2019). In the intestine, exposure to Wnt diminishes rapidly as cells migrate up the crypt–villus axis, where increasing concentrations of bone morphogenetic proteins are instead expressed, thus enabling exit from the cell cycle and the development of the differentiated cell phenotypes (Meyer et al, 2022).

For decades, the biology of the intestinal stem cells was considered analogous to that of well-studied hematopoietic stem cells, which undergo asymmetrical division to preserve the stem cell population and produce progenitor cells that differentiate into all other cell types comprising the hematopoietic system. Damage to the hematopoietic stem cell can be irreversible for the hematopoietic system—no other cell type can replace the stem cell if it is lost to disease.

Research over the last decade, however, shows that this model cannot be true for the intestine. When stem cells are lost in the severely damaged intestine, other differentiated cell types can revert to an undifferentiated state and repopulate the crypt base with LGR5 stem cells (Ayyaz et al, 2019; Brown et al, 2022; Viragova et al, 2024). This plasticity of well-differentiated epithelial cells, which enables them to rescue the stem cell niche, appears to occur in an ordered, preprogrammed fashion (Brown et al, 2022; Viragova et al, 2024)—starting with reversion to a fetal-like phenotype state associated with Yap/Taz-dependent transcriptional reprogramming and loss of the mammalian target of rapamycin complex 1 (mTORC1). Loss of mTORC1 activity triggers an autodegradative program that converts differentiated cell structure into building blocks supporting the change in phenotype (Willet et al, 2018), and then return of mTORC1 expression alongside a set of metaplastic genes for re-entry into the Wnt-dependent cell cycle. This ultimately leads to the rescue of the LGR5 stem cell phenotype—a process recently termed paligenosis (Brown et al, 2022; Willet et al, 2018).

The paper by Bracq et al in this issue of *EMBO Molecular Medicine* (Bracq et al, 2025) contributes meaningfully to our understanding of this process by elucidating the pathogenic mechanism underlying the protein-losing enteropathy in severe hyaline fibromatosis syndrome.

Hyaline fibromatosis syndrome is a rare autosomal disease caused by loss of function of the capillary morphogenesis gene 2 (CMG2). In all tissues, CMG2 regulates the composition of the extracellular matrix by post-translationally controlling collagen (and other hyaline proteins) expression via receptor-mediated endocytosis and lysosomal degradation (Burgi et al, 2017). The defect in this pathway largely explains the accumulation of excess extracellular hyaline material in tissues and the major clinical manifestations of the disease. But in severe forms of hyaline fibromatosis syndrome, children die from an intractable protein-losing enteropathy, the mechanism of which remains unclear.

Bracq and colleagues used mice lacking CMG2 (CMG2-KO mice) to study this issue. They found that under normal conditions the alimentary tract of CMG2-KO mice developed and functioned normally. Dysfunction only occurred after intestinal injury, which was chemically induced by exposure to dextran sulfate sodium (DSS). DSS induced the same level of intestinal damage in both wild-type (WT) and CMG2-KO mice. But unlike WT mice, the CMG2-KO mice failed to recover once exposure to DSS ceased.

**Fig. 1:**
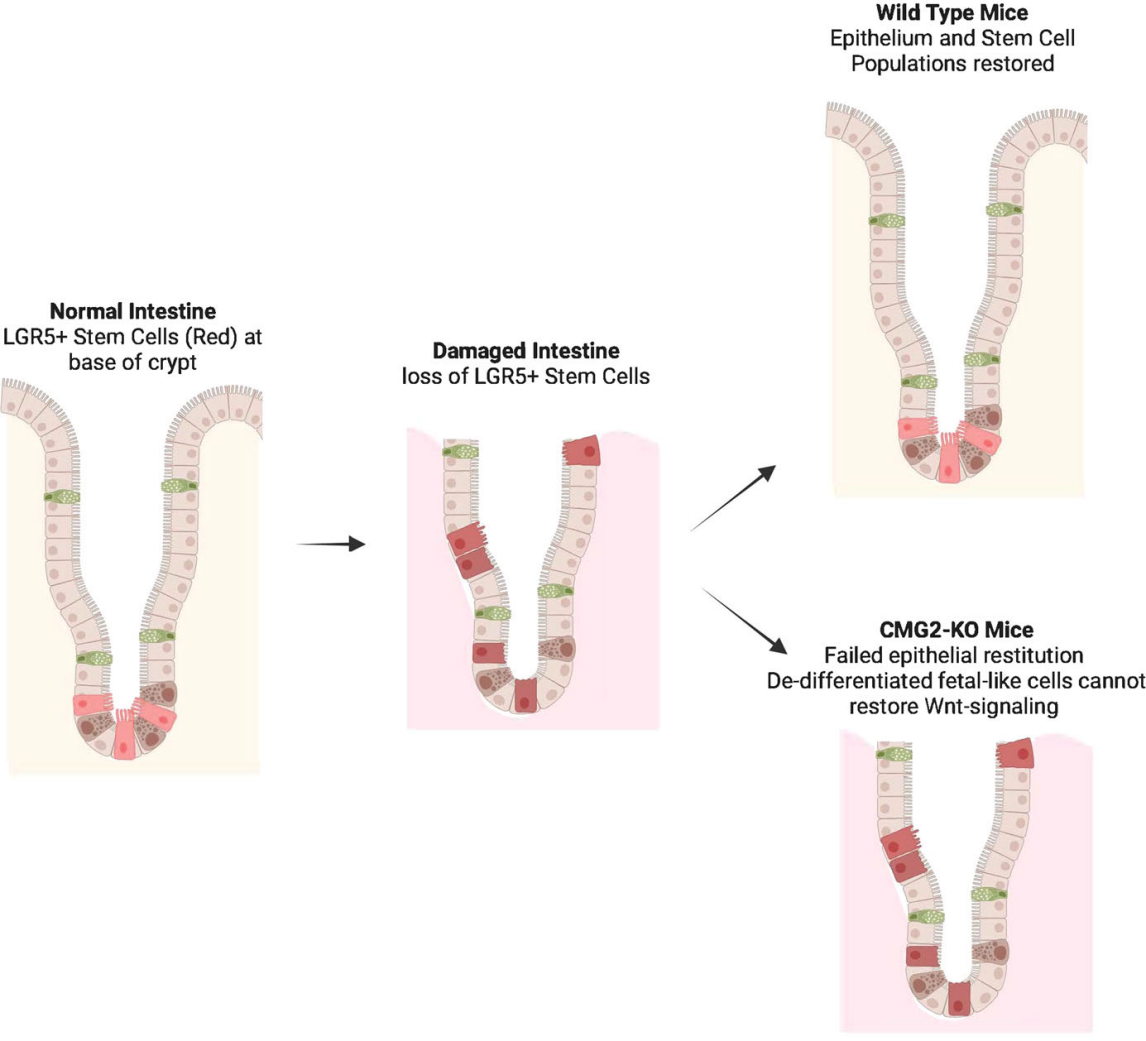
Recovery of intestinal LGR5+ stem cells (left panel, light red cells) when lost in damaged intestine (middle panel, damaged cells dark red) requires the expression of CMG2 for de-differentiated fetal-like cells to restore Wnt signaling and the cell cycle (right panels). Differentiated goblet cells are shown in green and Paneth cells in brown. Image created using Biorender.

Their study shows that in the mouse colon CMG2 plays a critical role in recovery from mucosal/epithelial damage when the epithelial stem-cell populations are depleted. Notably, the first anticipated step in the repair of such severe intestinal epithelial damage, whereby surviving differentiated epithelial cells are transformed back into a fetal-cell-like state, occurred normally in the CMG2-KO mouse. Rather, the block in restitution was found to occur at the step where the de-differentiated fetal-cell-like colonocytes should have been induced back into their WNT-dependent proliferative state—due to lack of CMG2 expression (Fig. 1). This finding is consistent with the dependence on CMG2 for WNT signaling in other experimental systems (Abrami et al, 2008). CMG2 acts on WNT signaling by forming a complex with lipoprotein-receptor-related protein 6 (Abrami et al, 2008; Wei et al, 2006), which assembles with Wnt-receptor Frizzled proteins to bind WNT ligands for signal transduction.

Notably, CMG2 did not affect the Wnt pathway during normal intestinal embryogenesis or in the maintenance of the LGR5+ stem cell progenitor compartment. Under non-stressed conditions, the guts of the CMG2-KO mouse were found to be normal. Hence, the notion that CMG2 must act in a context-specific way—after intestinal injury (Bracq et al, 2025).

On this account, one additional thought comes to mind. As CMG2 appears to affect Wnt signal transduction in an epithelial cell-autonomous way (see Bracq et al (2025)), the context-specific role of CMG2 in intestinal restitution may turn out to be explained by the rapidly diminishing concentration gradient of Wnt along the crypt-to-villus axis. In cases of severe intestinal damage where LGR5+ stem cells are lost, the absorptive and secretory cell types that undergo dedifferentiation to rescue the proliferative compartment must necessarily be located further up the crypt-to-villus axis, where Wnt concentrations are lower than at the crypt base. Thus, CMG2 may exert a context-specific physiological effect by amplifying the sensitivity of CMG2/Frizzled Wnt receptors in differentiated cells to low concentrations of Wnt.

The paper by Bracq et al makes a meaningful contribution to the field. It models an experiment of nature to test, delineate, and verify disease pathogenesis for hyaline fibromatosis syndrome, and it validates new ideas about the mechanisms of mucosal tissue regeneration after injury. These findings are likely to be applicable to tissue restitution beyond the intestine.
